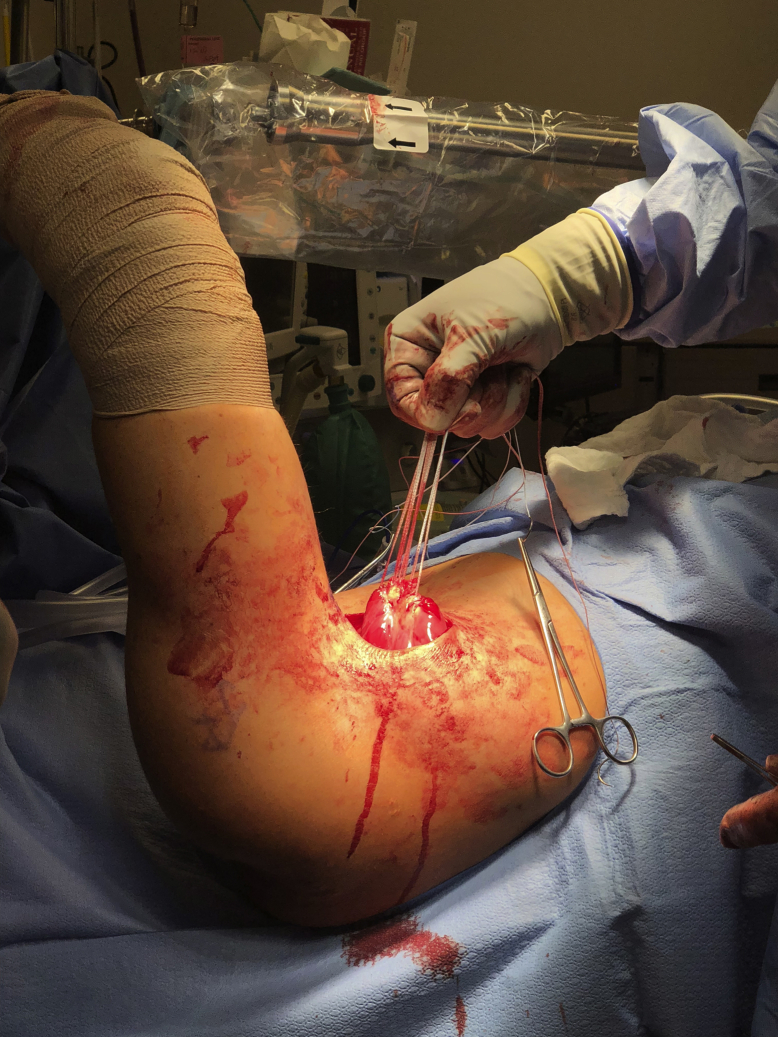# Single incision latissimus dorsi surgical technique: a three button repair

**DOI:** 10.1016/j.xrrt.2021.04.016

**Published:** 2021-05-24

**Authors:** J. Brett Goodloe, Kirsi S. Oldenburg, J. Mattison Pike, Josef K. Eichinger

**Affiliations:** Medical University of South Carolina, Charleston, SC, USA

**Keywords:** Latissimus dorsi, technique, three button, repair, anatomic reconstruction, tendon rupture

## Abstract

Due to the infrequent occurrence of latissimus dorsi insertional avulsions or tendon ruptures, there is no clear evidence on the optimal surgical fixation strategy. A three suture unicortical button repair technique through a single incision offers an anatomic reconstruction of the broad insertional footprint with adequate exposure. This fixation strategy is the preferred technique by the senior author.

Rupture of the latissimus dorsi insertion remains a relatively rare injury with minimal literature. As overhead athletes and power lifters continue to push the limits of explosive performance, added strain and potential injury exist. The decision for surgical management largely depends on functional demand, pain, and physical limitations that the patient experiences from the injury.^6^ This technique video demonstrates a single-incision approach with a three unicortical suture button technique which allows for anatomical repair of the broad insertional footprint of the latissimus dorsi tendon.

## Anatomy

**Figure 1:**
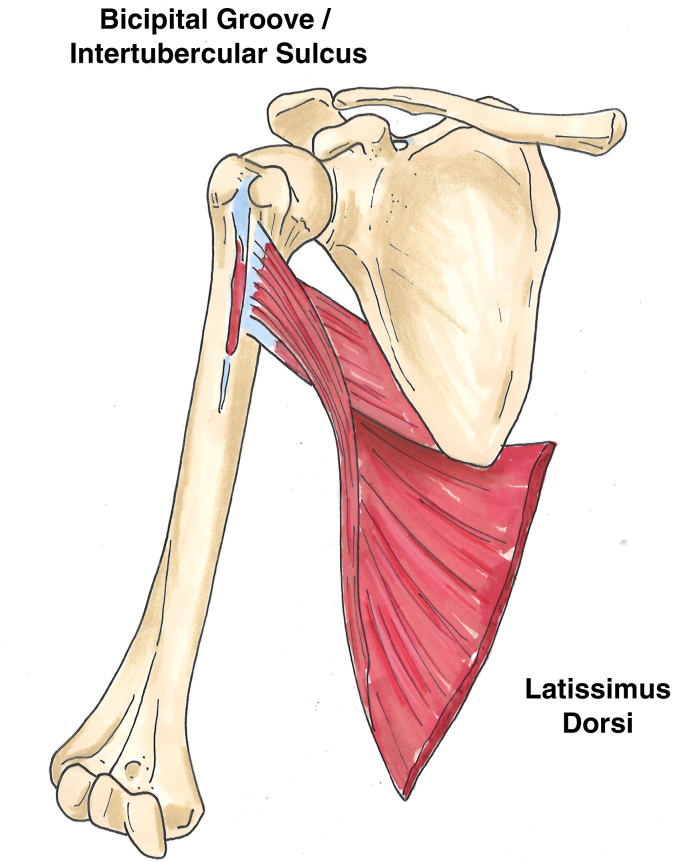
Anatomical drawing of the latissimus dorsi muscle and tendon.

The latissimus dorsi muscle contributes to thoracobrachial motion, including extension, adduction, and internal rotation of the arm. The muscle originates primarily from the thoracolumbar spine and coalesces into a tendon that inserts onto the floor of the intertubercular groove of the humerus (Fig. 1). A cadaveric study published by Pearle et al showed that the length of the latissimus dorsi tendon ranges from about 6 to 10 cm, and its width ranges from about 2 to 5 cm at its insertion.^9^

## Patient’s background

The patient is in his early 30’s and works as a law enforcement officer. He presented three weeks after a wake-surfing accident in which he sustained a traction type injury to his dominant arm. The injury resulted in complaints of pain in the periscapular region, with associated posterior axillary fold deformity and weakness with shoulder motion, and an inability to perform pullups.

## Preop evaluation

**Figure 2:**
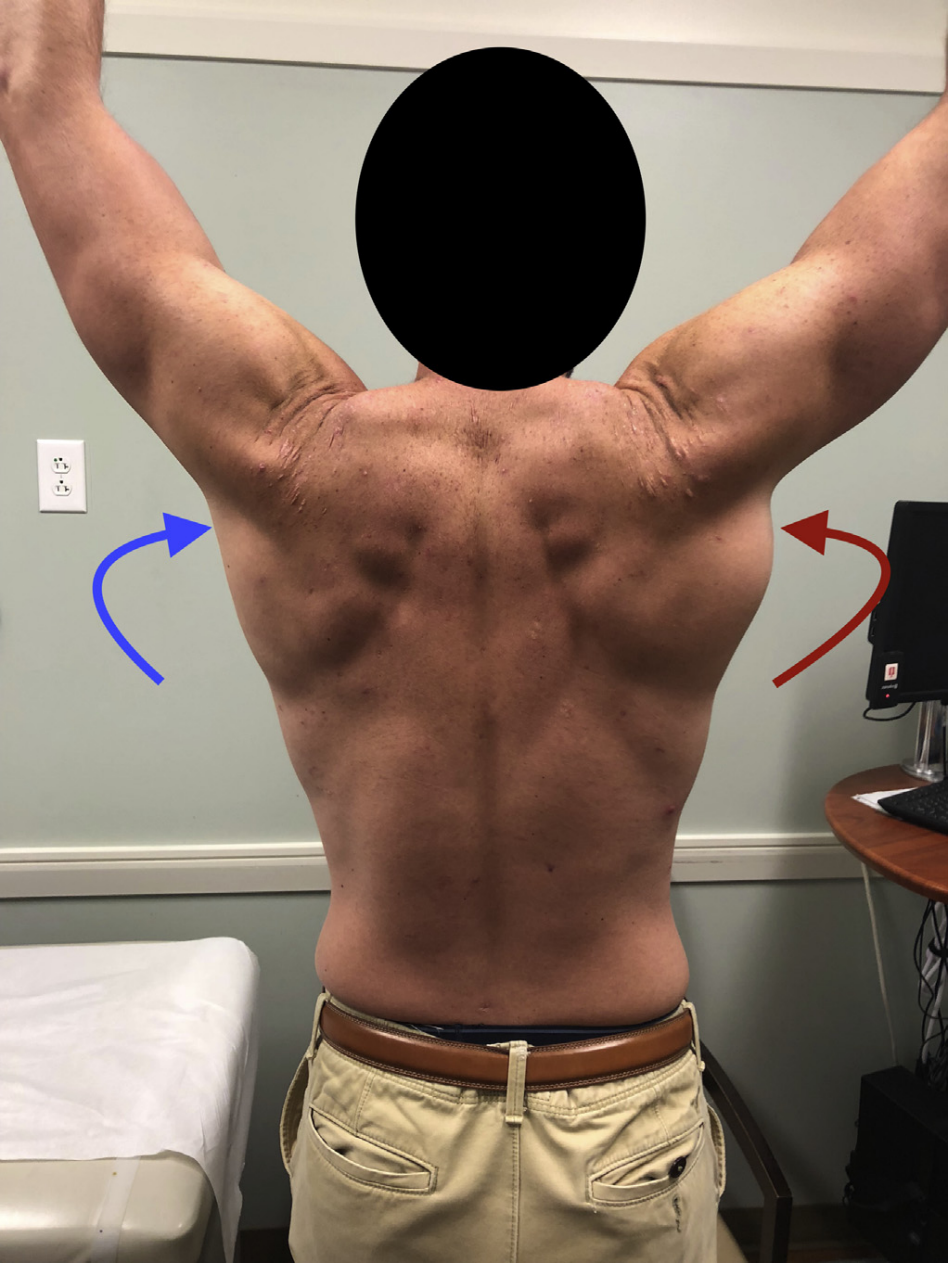
Preoperative imaging of the patient, demonstrating posterior axillary fold asymmetry with the  (red) pointing towards the patient’s injured side and the  (blue) pointing towards their non-injured side.

Visual inspection revealed right posterior axillary fold asymmetry (Fig. 2). Also present were swelling and ecchymotic changes in the axilla. Palpation along the posterior axillary fold and at the intertubercular groove revealed tenderness. The patient demonstrated weakness with adduction and extension indicative of a latissimus dorsi tendon tear. The patient otherwise demonstrated full active range of motion of the shoulder and a normal neurovascular examination.

## Magnetic resonance imaging

**Figure 3:**
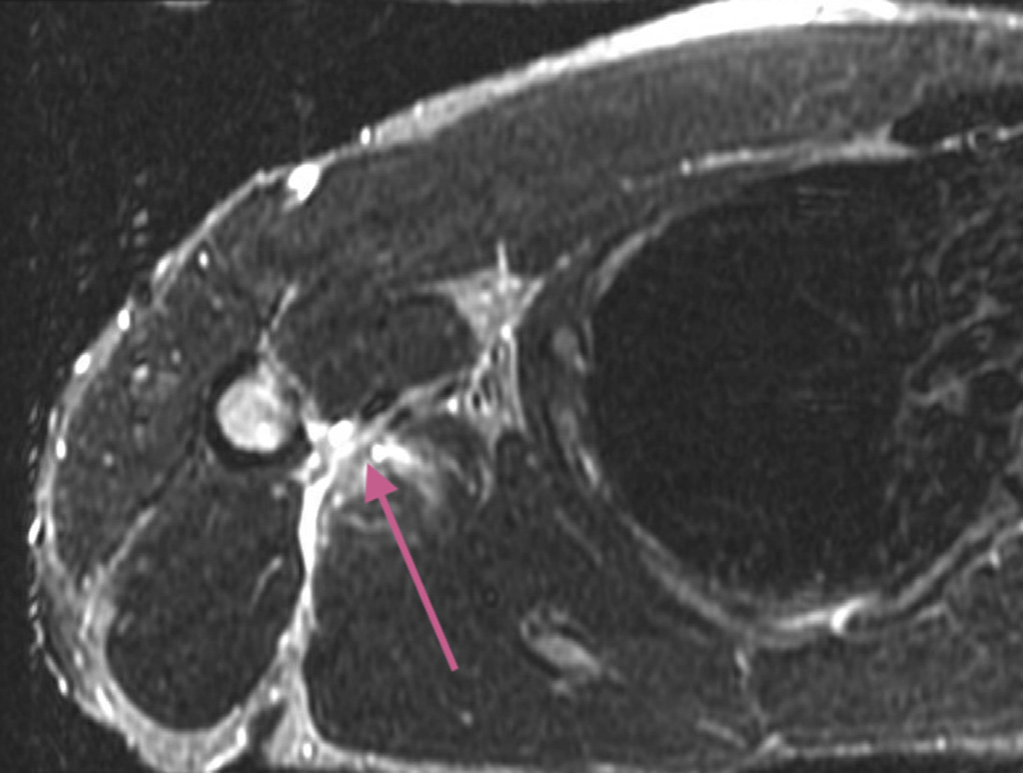
T2 axillary sequence with the  pointing towards the increased signal adjacent to the intertubercular groove.

**Figure 4:**
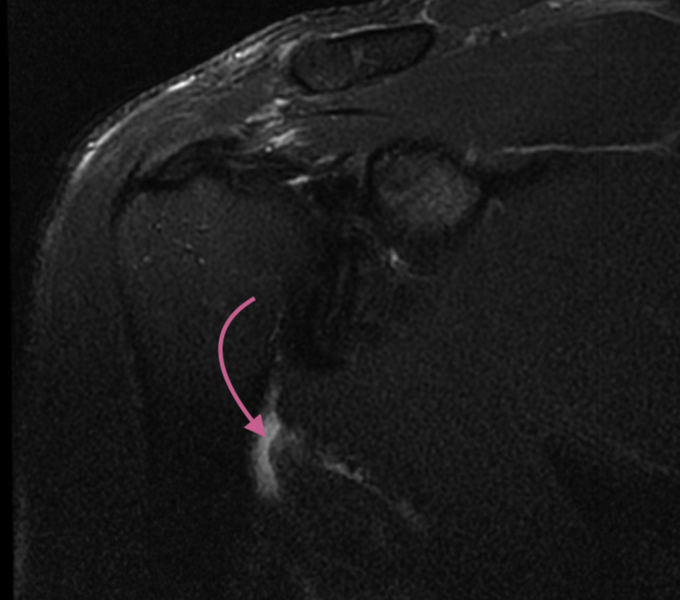
T2 Fat Sat coronal imaging with the stump of the retracted tendon being pointed at by .

A standard magnetic resonance imaging (MRI) of the shoulder can visualize the torn tendon. As seen on the T2 axillary sequences (Fig. 3), increased signal adjacent to the intertubercular groove highlights the tear and the associated inflammation. The T2 Fat Sat coronal imaging similarly reveals a complete tear of the lattisimus dorsi tendon (Fig. 4). An MRI of the thorax and involved arm and shoulder is recommended for tear assessment if there is substantial retraction. MRI’s of the thorax with bilateral humerii will allow for control comparison to the noninjured extremity. The authors recommend treating physicians to specify the latissimus tendon as the area of injury to the radiologists and technicians in addition to utilizing an MRI that extends distally to better visualize the area of interest.

## Surgical technique

**Figure 5:**
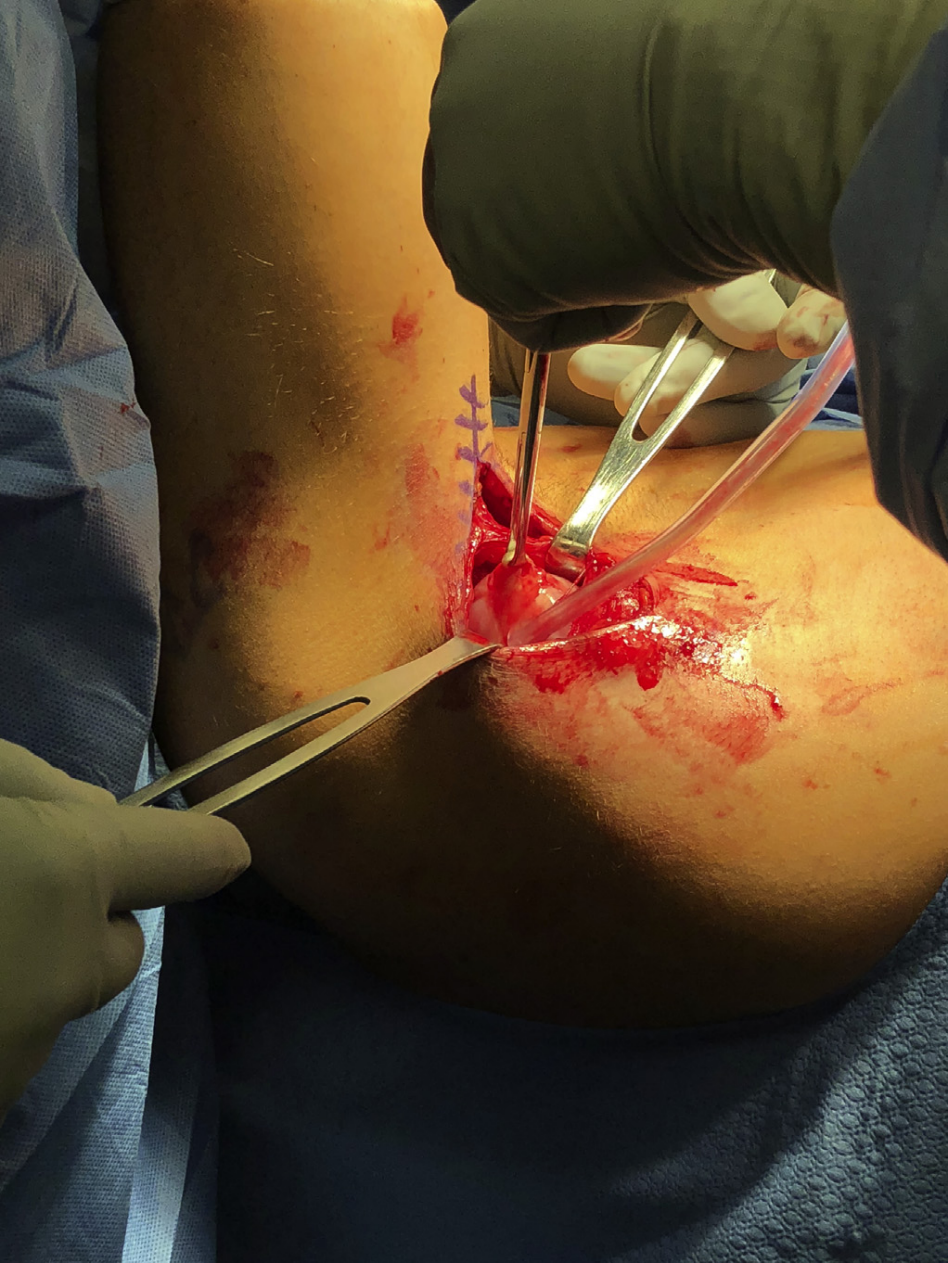
Identification of the tendon is followed by blunt dissection of the humeral insertion site and the tendon is grabbed with Allis clamps.

**Figure 6:**
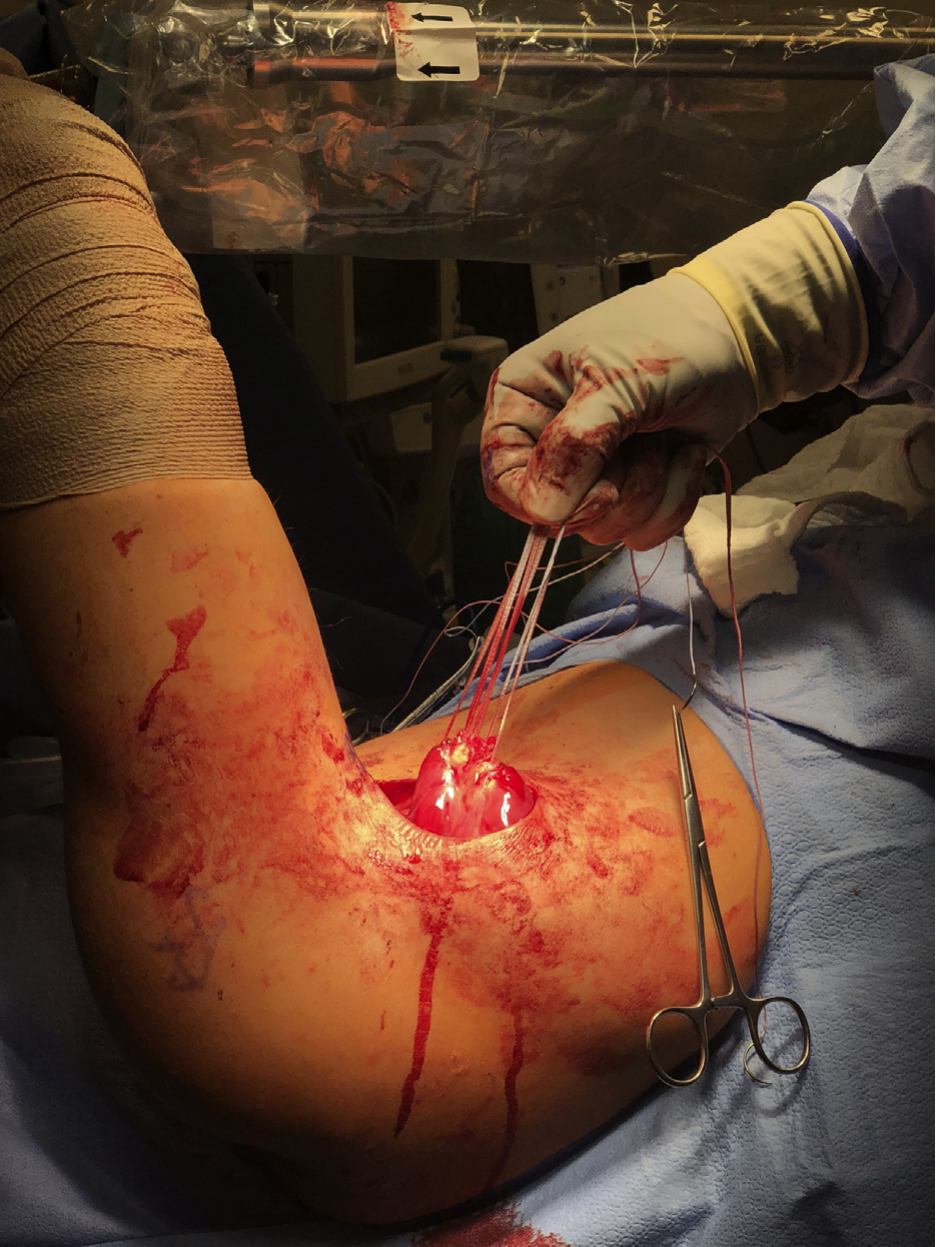
Mobilization of the muscle/tendon unit is performed.

**Figure 7:**
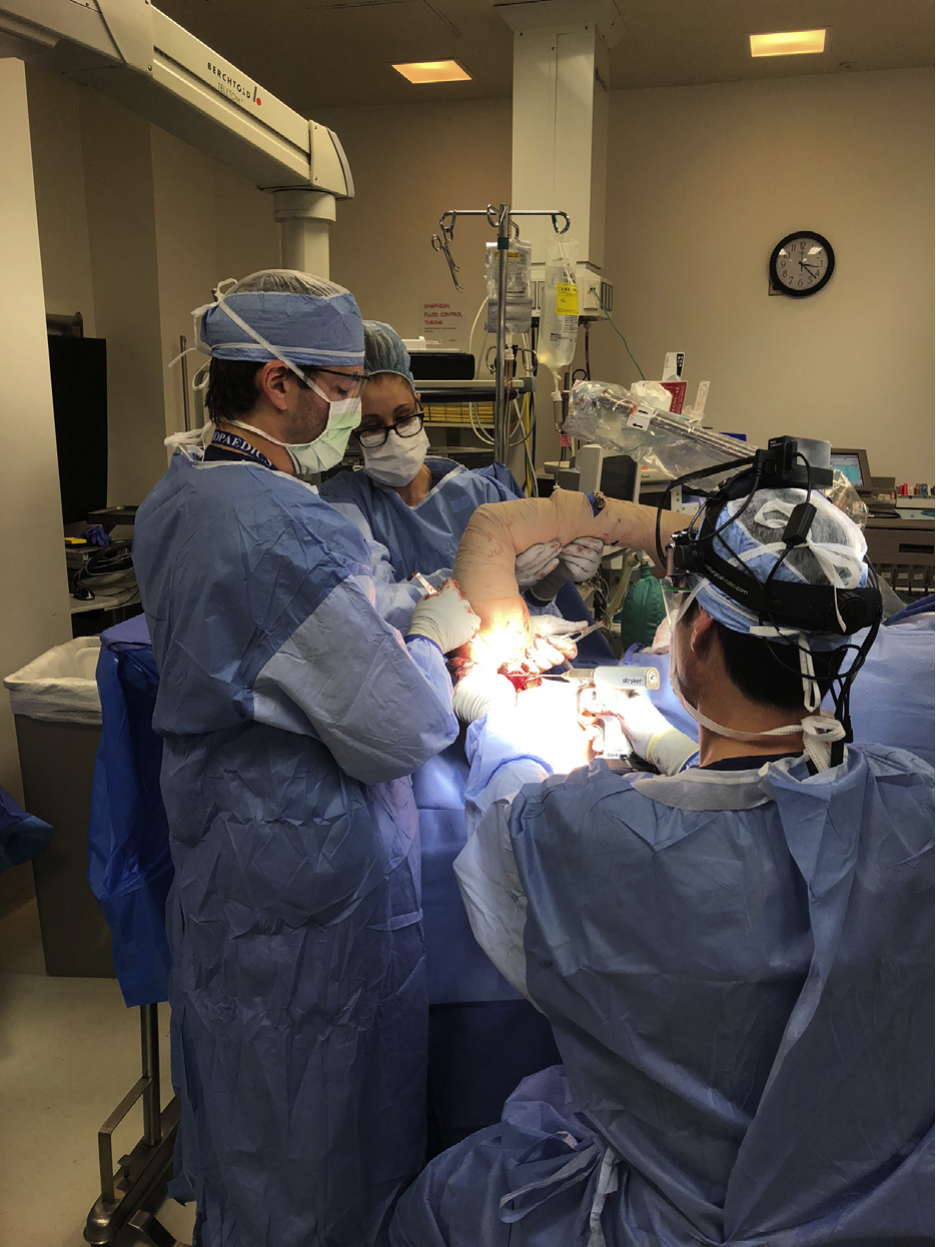
Three parallel unicortical drill holes are placed at the insertional site.

The patient is positioned in the lateral decubitus position on a padded pegboard with an axillary roll. The bed is turned 90 degrees so that the patient faces the anesthesia team (Video 1). The operative upper extremity is widely prepped, draped with split drapes, and placed in a dynamic limb positioner. The limb is then flexed, adducted, and internally rotated to allow for full visualization of the posterior axillary fold. The skin is marked along the posterior axillary fold to the level of the proximal humeral shaft. An 8-cm incision is made along the marking with the arm in an adducted, flexed and internally rotated position. Blunt dissection is used to develop fascial planes overlying the latissimus and sharp dissection is used to locate the latissimus tendon. The ruptured tendon is often encountered with a rush of serous fluid (Fig. 5). Evaluation of tear characteristics is performed to confirm if a humeral avulsion versus myotendinous junction tear occurred. Depending on the chronicity or degree of retraction, the tendon must be mobilized to allow for excursion for successful repair back to the native insertion site. It is the author’s preference to utilize three FiberTape sutures (Arthrex, Naples, FL, USA) due to the broad and flat tendon characteristics. Sutures are passed in a running-locking fashion to secure the latissimus tendon. Tendon mobilization confirms appropriate excursion to the humeral insertion site (Fig. 6). To expose the humeral shaft, two army-navy retractors are placed on the undersurface of the triceps. This maneuver also protects the posterior cutaneous brachial nerve from iatrogenic injury with the drill. Depending on visibility, the operative arm can be positioned using the dynamic limb positioner or can be repositioned by removing the hand from the dynamic limb positioner and placing it in position for visualization of the humeral footprint. Debridement of the humeral footprint can be conducted with a curette or rongeur as needed. Three, parallel unicortical drill holes are placed at the insertion site to maximize the width of the tendon and recreate the true anatomical insertion (Fig. 7). The suture buttons are loaded in a stepwise fashion using the tension slide technique and are inserted into the unicortical holes from proximal to distal. The buttons are flipped and the sutures are tensioned and tied for added fixation. Range of motion of the shoulder is performed to assess the tension of the repair. The incision is closed with buried interrupted absorbable sutures reapproximating the deep fascial layer, followed by deep dermal layer closure with interrupted absorbable suture and finally, subcuticular layer closure with a running absorbable suture.

## Postoperative rehabilitation

Postoperatively, the patient is placed in an abduction shoulder brace. At four weeks, the patient begins active range of motion with physical therapy supervision. Range of motion exercises for the hand, wrist, and elbow, in addition to pendulum exercises, are initiated on postoperative day 1. At 4 weeks, the patient begins active range of motion exercises with physical therapy supervision. Substantial strengthening activities are initiated at a minimum of 12 weeks from surgery.

## Postoperative clinical assessment

In this case, the patient's clinical examination at 8 months postsurgery revealed restoration of the anatomy of the posterior axillary fold and symmetric muscular appearance. His healed scar was hidden in the posterior axilla for an optimal cosmetic result. At one year postsurgery, the patient reported a subjective shoulder value of 100% with no pain and restoration of all functional activities without limitations.

## Pearls and pitfalls

When using this technique, careful dissection should prevent iatrogenic injury to the posterior cutaneous nerve branch. Appropriately mobilizing the latissimus tendon and confirming appropriate excursion to the humeral insertion is important prior to fixation. The operating surgeon can remove the operative limb from the dynamic limb positioner to ensure appropriate drill placement and simplify positioning. Additionally, suture fixation in the latissimus tendon prior to reinsertion deep into the humeral insertion site allows for easy passage of sutures. It is important to space the unicortical buttons along the insertional footprint as drilling too close to the adjacent button may weaken the interposed cortex. Placing the unicortical buttons at the most proximal and distal aspect of the insertional site should distribute force upon tendon activation.

## Discussion

Latissimus dorsi tendon ruptures are rare injuries with limited literature. The indications for surgery remain largely unknown, with surgical repair or reconstruction generally being determined based on the degree of tendinous involvement, the degree of retraction present, and the functional necessity required for sport or labor. The current recommendation for partial tears is conservative management with acute rest followed by gradual rehabilitation, with Friedman et al demonstrating relatively successful rehabilitation of a CrossFit athlete using conservative methods.^5^ The overall suture anchor repair literature, regardless of surgical technique, has been excellent with a high percentage of patients returning to full activity.^6^

The senior author prefers a small posterior axillary single incision approach comparable to previously described single incision techniques.^1^^,^^7^^,^^8^ This incision combined with lateral decubitus positioning provides excellent exposure of the retracted tendon and the humeral insertion. Direct exposure of the humerus can be achieved with a relatively small incision which allows for appropriate intraoperative adjustments, easy identification of the entirety of the humeral insertion, and appropriate tensioning assessment after repair. In addition, applying the suture fixation with a free needle prior to suture button placement allows for secure fixation and simplifies suture passage. Also, utilization of three suture buttons allows for appropriate spread of the tendinous insertion to recreate the natural anatomy, especially in larger stature patients.

The senior author’s preferred technique differs from other recently published surgical technique case reports. The suture button technique is similar to a recently published technique by Holschen et al who described a single incision laterally positioned technique with only a two-button monocortical fixation repair.^7^ Holschen et al preferred button fixation, as opposed to anchor or biotenodesis screw fixation, as they felt it provided ample fixation and visibility for radiographic confirmation of appropriate button placement. Their study reported an excellent outcome in a CrossFit Athlete.^7^ The wide three points of fixation preferred by the senior author of this report are similar to the 3-point bony fixation proposed in a case study by Ellman et al.^4^ Ellman et al performed a three suture anchor fixation with two double-loaded suture anchors both superiorly and inferiorly with a central metallic corkscrew anchor. Their procedure was performed through a dual incision approach with patient repositioning.^4^

## Conclusion

We believe that three suture buttons provides the greatest anatomical fixation and helps distribute force. Unicortical button fixation eliminates the risk of iatrogenic injury via bicortical button fixation and provides similar, if not better biomechanical properties to interference screw fixation in regards to subpectoral biceps tenodesis.^2^^,^^3^ Additionally, the senior author believes that a three button repair provides enough solid fixation to allow for earlier range of motion activity of the shoulder joint to prevent stiffness, although, as with other described rehabilitation programs, return to strengthening and sport participation does not begin prior to 3 months.

## Disclaimers

Funding: No funding was disclosed by the author(s).

Conflicts of interest: The authors, their immediate families, and any research foundations with which they are affiliated did not receive any financial payments or other benefits from any commercial entity related to the subject of this article.

## Figures and Tables

**Video Still:**